# Community-based MDR-TB care project improves treatment initiation in patients diagnosed with MDR-TB in Myanmar

**DOI:** 10.1371/journal.pone.0194087

**Published:** 2018-03-29

**Authors:** Pyae Phyo Wai, Hemant Deepak Shewade, Nang Thu Thu Kyaw, Saw Thein, Aung Si Thu, Khine Wut Yee Kyaw, Nyein Nyein Aye, Aye Mon Phyo, Htet Myet Win Maung, Kyaw Thu Soe, Si Thu Aung

**Affiliations:** 1 International Union against Tuberculosis and Lung Disease (The Union), Mandalay, Myanmar; 2 International Union against Tuberculosis and Lung Disease (The Union), South-East Asia Office, New Delhi, India; 3 National Tuberculosis Programme, Ministry of Health and Sports, Mandalay, Myanmar; 4 National Tuberculosis Programme, Ministry of Health and Sports, Nay Pyi Daw, Myanmar; 5 Department of Medical Research (Pyin Oo Lwin Branch), Ministry of Health and Sports, Pyin Oo Lwin, Myanmar; Central University of Tamil Nadu, INDIA

## Abstract

**Background:**

The Union in collaboration with national TB programme (NTP) started the community-based MDR-TB care (CBMDR-TBC) project in 33 townships of upper Myanmar to improve treatment initiation and treatment adherence. Patients with MDR-TB diagnosed/registered under NTP received support through the project staff, in addition to the routine domiciliary care provided by NTP staff. Each township had a project nurse exclusively for MDR-TB and 30 USD per month (max. for 4 months) were provided to the patient as a pre-treatment support.

**Objectives:**

To assess whether CBMDR-TBC project’s support improved treatment initiation.

**Methods:**

In this cohort study (involving record review) of all diagnosed MDR-TB between January 2015 and June 2016 in project townships, CBMDR-TBC status was categorized as “receiving support” if date of project initiation in patient’s township was before the date of diagnosis and “not receiving support”, if otherwise. Cox proportional hazards regression (censored on 31 Dec 2016) was done to identify predictors of treatment initiation.

**Results:**

Of 456 patients, 57% initiated treatment: 64% and 56% among patients “receiving support (n = 208)” and “not receiving support (n = 228)” respectively (CBMDR-TBC status was not known in 20 (4%) patients due to missing diagnosis dates). Among those initiated on treatment (n = 261), median (IQR) time to initiate treatment was 38 (20, 76) days: 31 (18, 50) among patients “receiving support” and 50 (26,101) among patients “not receiving support”. After adjusting other potential confounders (age, sex, region, HIV, past history of TB treatment), patients “receiving support” had 80% higher chance of initiating treatment [aHR (0.95 CI): 1.8 (1.3, 2.3)] when compared to patients “not receiving support”. In addition, age 15–54 years, previous history of TB and being HIV negative were independent predictors of treatment initiation.

**Conclusion:**

Receiving support under CBMDR-TBC project improved treatment initiation: it not only improved the proportion initiated but also reduced time to treatment initiation. We also recommend improved tracking of all diagnosed patients as early as possible.

## Introduction

Multidrug-resistant/Rifampicin-resistant tuberculosis (MDR-TB/RR-TB) poses a major threat to the control of tuberculosis globally with an estimated 580,000 cases in 2015. However, there are gaps in MDR-TB diagnosis and treatment initiation in many of the high MDR-TB burden countries [1]. The gap (called as pre-treatment loss to follow-up) and delay in treatment initiation can lead to pre-treatment mortality and possibly poor MDR-TB treatment outcomes: fueling the further transmission of MDR-TB.

The pre-treatment loss to follow-up in patients with MDR-TB was about 21% in Bangladesh, 39% before implementation of Line Probe Assay (LPA) and 12% after implementation of LPA in India and 55% in South Africa [2–4]. In 2016, a systematic review identified no published evidence linking delay in treatment initiation to MDR-TB outcomes [5]. Recently, a study from India has reported delayed treatment initiation (>30 days) as a risk factor for unfavorable outcomes [6].

Myanmar is one of the 30 high MDR-TB burden countries in the world [7]. The National Tuberculosis Programme (NTP) is committed to scale up the case detection of MDR-TB through implementation of Xpert MTB/Rif starting from 2011. The treatment and care of MDR-TB is domiciliary and provided according to World Health Organization (WHO) recommended programmatic management of DR-TB (PMDT) model since 2011[8]. Baseline investigations and treatment initiation are done at MDR-TB treatment centers followed by domiciliary care (directly observed treatment–DOT) in the community.

In 2014, WHO estimated that there were 9,000 MDR-TB cases in Myanmar and 5,500 cases were estimated by NTP. In 2015, NTP reported that 2,793 MDR-TB cases were diagnosed and 2,207 were enrolled for treatment in the same year [7,9]. The gap between notified and treated MDR-TB cases in Myanmar was reported as 61% and 43% in 2013 and 2014 respectively [9]: some patients were possibly dying before getting treated.

By 2020, Myanmar targets to enroll all MDR-TB patients on treatment within two weeks of their diagnosis and provide comprehensive patient support package to enable treatment success rates of >80% [7]. To achieve this, the International Union Against Tuberculosis and Lung Disease (The Union) in collaboration with NTP started the community-based MDR-TB care (CBMDR-TB Care) project in upper Myanmar since 2015 with funding from Global Fund (GF) and Three Millennium Development Goal Fund (3 MDGF). Patients with MDR-TB diagnosed/registered under PMDT received support through the project staff, in addition to the domiciliary care provided by NTP’s PMDT staff. Trained community volunteers and project focal nurses (exclusively for MDR-TB) provided psychosocial and socio-economic support to patients and family members after MDR-TB diagnosis up to treatment initiation and completion under the guidance of NTP township TB team.

Phase-wise implementation of project in Upper Myanmar between 2015 and 2016 provided us a unique opportunity to assess the impact of this project. There is no published literature on interventions to improve treatment initiation among MDR-TB through a support package (CBMDR-TBC in our case) in the context of domiciliary care through PMDT. Therefore, as a first ever study, we aimed to assess whether the Union’s CBMDR-TBC project improved treatment initiation in patients diagnosed with MDR-TB. The effect of CBMDR-TBC project on unfavourable outcomes and death among registered patients during initial 8 months of treatment is published elsewhere [10].

## Methods

### Study design

This was a retrospective cohort study involving record review.

### Setting

#### General setting

Myanmar is a lower middle income country [11] in south-east Asia region flanked by India and Bangladesh in north-west; China in north-east; Laos and Thailand in south-east; Bay of Bengal in south-west and Andaman sea in south. It has a population of 51 million and predominantly mountainous in upper Myanmar, plain and delta region in middle and lower Myanmar [12]. It is administratively divided into states/regions (n = 15) followed by districts (n = 67) and townships (n = 330). Under the National Tuberculosis Program, there are TB centers at central level and systematically decentralized to state /region level, district level and township level [13].

#### Programmatic management of drug-resistant tuberculosis (PMDT) in Myanmar

Patients with presumptive MDR-TB are referred from the township TB center to the nearest district TB center with Xpert MTB/Rif diagnostic facility. Each Xpert MTB/Rif facility has a laboratory register. A line list of presumptive MDR-TB register is maintained at the township level.

All patients diagnosed as RR-TB by Xpert MTB/RIF are assumed as MDR-TB and started on second line treatment immediately. In select cases (presumed to be having a low-risk of MDR-TB), an initial positive result is reconfirmed by a repeat Xpert MTB/RIF. If needed, final confirmation is done with line probe essay (LPA) or culture and drug susceptibility test (DST) using MGIT (Mycobacteria growth indicator tube) liquid system [7]. The LPA and MGIT register are kept at upper Myanmar TB center in Mandalay.

After the patient is diagnosed with MDR-TB, the respective township TB team is informed. The township TB team includes township medical officer, township TB coordinator, basic health staff (BHS) and laboratory technician. The support package given by the BHS under PMDT to ensure treatment initiation is shown in **Table 1**. The BHS is also involved in implementing activities under other national health and disease control programmes. After the patient principally agrees to undertake MDR-TB treatment and informs to make clear that he/she will take DOT for at least 20 months, the patient is referred to the nearest MDR-TB treatment center (district-level). After pre-treatment evaluation which includes baseline investigations and measurements, the patient is initiated on treatment. MDR-TB treatment card and register are maintained at the MDR-TB treatment center and patient has a treatment booklet. All services are provided free of cost (7).

**Table 1:** Package of support to patients diagnosed with MDR-TB for treatment initiation by NTP’s PMDT in Myanmar, 2015–16 [7].

1	Initial home visit and pretreatment counselling including the nature of medicines to be taken, the treatment process and the necessity of directly observed treatment to monitor the treatment and offer regular support by the township medical officer, township TB coordinator and basic health staff
2	Base-line investigations at the MDR-TB treatment center
3	Transport of sputum to the MDR-TB treatment center for diagnosis confirmation, if required

TB–Tuberculosis; MDR-TB–multi drug resistant tuberculosis

#### Community-based MDR-TB care (CBMDR-TBC) project

**Fig 1:**
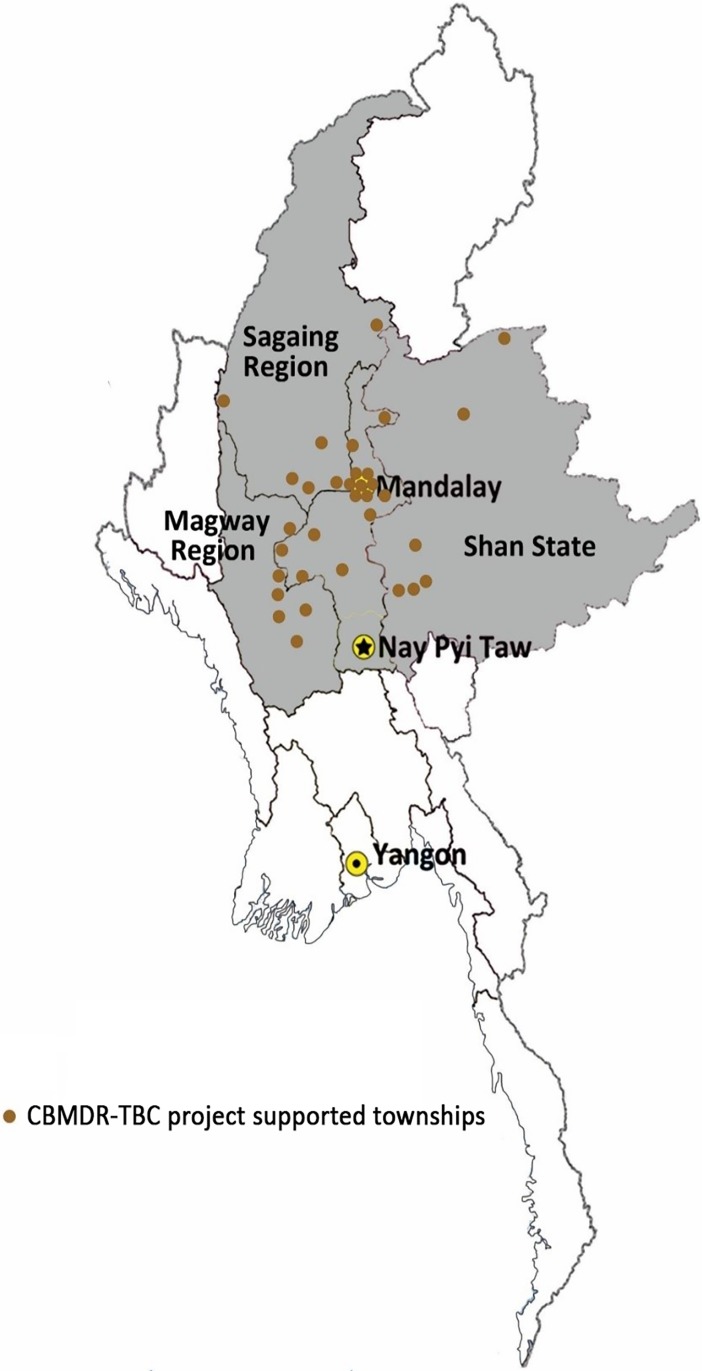
Map of Myanmar showing 33 CBMDR-TBC project supported townships across four states/regions of upper Myanmar, 2015–16. *CBMDR-TBC project–community-based multi-drug resistant tuberculosis care project.

In order to increase treatment initiation and treatment adherence, CBMDR-TBC project supports PMDT in 33 townships (selected after consultation with NTP), across four states/regions in upper Myanmar since January 2015 **(Fig 1).** The project was implemented phase wise across these 33 townships between January 2015 and June 2016. Once the project implementation began in a particular township, all old diagnosed/treated patients and newly diagnosed cases were provided support. The support is provided from diagnosis to treatment initiation, up to treatment completion.

Each project township has a project focal nurse under supervision of the township TB team from NTP. In addition to implementation of the care package under PMDT (**Table 1),** the project focal nurse also identifies and trains a volunteer who stays close to the patient in the community. The complementary support provided from diagnosis to treatment initiation under CBMDR-TBC is summarized in **Table 2**.

**Table 2:** Support package to all MDR-TB patients from diagnosis to treatment initiation under the community-based MDR-TB care (CBMDR-TBC) project in Myanmar, 2015–16.

1	Exclusive project focal nurse at each project township has to support following
	Initial home visit and pretreatment counselling
	Psychosocial counselling to patient and family members by the project focal nurse
	Coordinate to undergo base-line investigations at MDR-TB treatment center
	Recruited and trained volunteer for evening DOT once patient starts treatment
	Community mobilization by providing health education to the patient and their family members and neighbours
2	Pre-treatment support: 30 USD per month for a maximum of four months for patients with intent to reduce, to some extent, their expenses in lodging, some ancillary drugs

MDR-TB–multi drug resistant tuberculosis, DOT–directly observed treatment; USD–United States Dollars

During 2015–16, of 49 Xpert MTB/Rif machines in the country, 12 were in 33 CBMDR-TBC townships. Of the 73 MDR-TB treatment centers in Myanmar, 15 were in 33 CBMDR-TBC project townships.

Routine monitoring includes submission of monthly reports by volunteers to project focal nurse and then by the project focal nurses to project manager (one manager is assigned for every eleven townships) which are then forwarded to the monitoring and evaluation unit of The Union Office and the township TB team under NTP.

### Study participants

All patients diagnosed with MDR-TB between January 2015 and June 2016 in 33 CBMDR-TBC project townships of upper Myanmar were included. Date of diagnosis was the entry date into the cohort, while date of treatment initiation or date of censoring (31 Dec 2016), whichever was earlier, was the end-date in the cohort.

### CBMDR-TBC exposure ascertainment

To study the effect of CBMDR-TBC project on treatment initiation, we categorized the patients into two groups: ‘receiving support’, and ‘not receiving support’ using the date of diagnosis and date of project initiation in patient’s township. ‘Receiving support’ included patients who received support from CBMDR-TBC project from the date of MDR-TB diagnosis (date of project initiation in patient’s township was on or before date of diagnosis). Other patients were classified as ‘not receiving support’.

### Data variables, sources of data and data collection

Between January and March 2017, records of all Xpert MTB/Rif, LPA and MGIT tested positive patients were extracted from the 12 Xpert MTB/Rif facility laboratory registers and upper Myanmar TB center Laboratory unit located in Mandalay. After removal of duplicates each study participant was given a unique identifier which was a combination of Xpert MTB/Rif facility code, Xpert MTB/Rif laboratory number and year. Date of diagnosis was defined as the date of Xpert MTB/Rif, LPA or MGIT test results. Earlier date was used in case of more than one test results.

Variables collected from laboratory register were age, sex, region, previous treatment history of TB and HIV status. Distance between patient’s resident Township and MDR-TB treatment center was calculated using google maps (www.googlemaps.com). Date of project initiation in patient’s township was collected from CBMDR-TBC project records.

Treatment initiation was confirmed by matching the name, age and resident township (if unique identifier details were not available) in the MDR-TB treatment register. Variables collected were treatment initiation (yes/no) and date of treatment initiation.

### Analysis and statistics

Data collected in structured data collection forms were single entered into EpiData (Version 3.1, EpiData Association, Odense, Denmark) at each MDR-TB treatment center by research assistants between March and April 2017. Descriptive analysis (frequency, proportion, means (SD), median (IQR)) and generation of derived variables was done using EpiData analysis (version 2.2.2.183, EpiData Association, Odense, Denmark). STATA (version 12.1, copyright 1985–2011 StataCorp LP USA, serial number: 30120504773) was used for time to event (treatment initiation) analysis and multivariable predictive modelling.

‘Receiving support’ was the exposure of interest. Treatment initiation was the outcome of interest which was summarized as proportions and incidence rate (number of events per 1000 person-days of follow-up).

Unadjusted analysis was done to determine the association (Hazard ratio, HR) between “receiving support”, other potential confounders and treatment initiation. Cumulative proportion (1- Kaplan-Meier) of treatment initiation was described over time: overall and by CBMDR-TBC status. For independent predictors of treatment initiation, age, sex, CBMDR-TBC status and variables with *p*-value of <0.2 in the unadjusted analysis were included (after ruling out multi-collinearity) in the Cox regression model (enter method). We assessed for proportional hazard assumption of the model by using Schoenfeld residuals and plotting the estimated survival curves using Cox model and Kaplan-Meier estimates. We modelled time-varying covariates (using tvc function in STATA) in case the proportional hazard assumption was not met [14]. Unadjusted and adjusted HRs were reported with 95% confidence intervals (CI).

### Ethics and consent

Ethics approval was received from Myanmar Ethics Review Committee, Department of Medical Research, Ministry of Health and Sports, Myanmar (ERC No. 014216, dated 30^th^ January 2017) and the Ethics Advisory Group of International Union against Tuberculosis and Lung Disease (The Union), Paris, France (EAG No. 81/16, dated 1^st^ November 2016.) Permission to conduct the study was granted from National Tuberculosis Programme, Ministry of Health and Sports, Myanmar. As the study involved analysis of secondary data from programme records, waiver for informed consent was sought and approved by the ethics committees.

## Results

### Baseline characteristics

There were 456 patients diagnosed with MDR-TB, 208 (46%) were “receiving support” and 228 (50%) were “not receiving support” from CBMDR-TBC project. CBMDR-TBC status in 20 (4%) patients was not known because of missing diagnosis dates. The mean (SD) age of patients was 40 (15) and 305 (67%) were male. More than half of them were from Mandalay region (n = 277). Seventy nine percent (n = 359) had previous history of TB and 29% (130) of them were residing in townships >100 kilometer away from the MDR-TB treatment center. **(Table 3)**

**Table 3:** Baseline characteristics of patients diagnosed with MDR-TB between January 2015 and June 2016 in 33 CBMDR-TBC project supported townships in Myanmar.

Characteristics		n	(%)
**Total**		**456**	**(100)**
Age (year)			
	< 15	4	(1)
	15–34	187	(41)
	35–54	178	(39)
	≥55	87	(19)
Sex			
	Male	305	(67)
	Female	151	(33)
Patient residence state/region			
	Mandalay	277	(61)
	Magway	41	(9)
	Sagaing	62	(14)
	Northern Shan	49	(11)
	Southern Shan	27	(6)
Previously treated TB			
	Yes	359	(79)
	No	86	(19)
	Unknown	11	(2)
HIV status			
	Non-reactive	255	(56)
	Reactive	127	(28)
	Unknown	74	(16)
Distance from treatment facilities			
	Same township	114	(25)
	<100 km	212	(47)
	≥ 100 km	130	(29)
Under CBMDR-TB Care project*			
	No	228	(50)
	Yes	208	(46)
	Unknown	20	(4)

MDR-TB—Multi drug resistant tuberculosis

*Patient considered under CBMDR-TB Care project if date of project initiation in patient’s township was before the date of MDR-TB diagnosis, date of MDR-TB diagnosis not available for 20 patients and therefore could not be classified

### Treatment initiation

There were 96047 person-days of follow: it was 64766 and 31281 among “not receiving support” and “receiving support” group respectively.

Overall, 57% (261/456) patients initiated treatment. Among those initiated on treatment (n = 261), only 14% (36/261) and 38% (99/261) initiated treatment within 14 and 30 days respectively. Thirty five patients initiated treatment after 100 days of diagnosis and twelve initiated treatment after 200 days.

**Fig 2:**
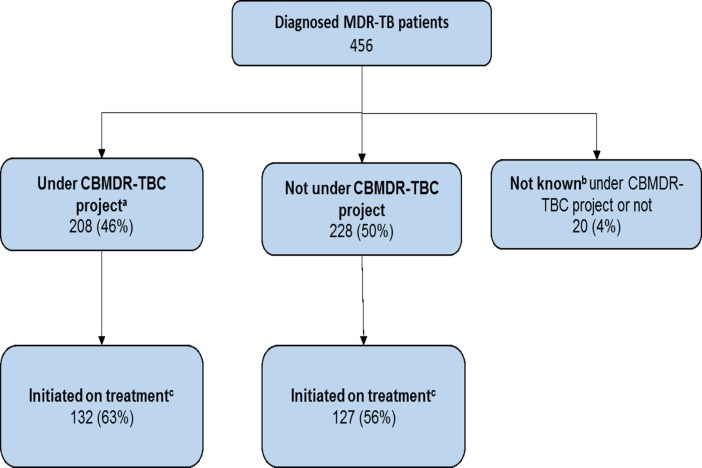
Flow chart showing treatment initiation cascade stratified by CBMDR-TBC status among diagnosed MDR-TB patients in 33 CBMDR-TBC project supported townships of upper Myanmar, January 2015-June 2016. *MDR-TB: Multi drug resistant tuberculosis^. a^Patient considered receiving support if date of project initiation in patient’s township was before the date of MDR-TB diagnosis, date of MDR-TB diagnosis is missing for 20 patients and therefore could not be classified. ^b^whether patients were under CBMDR-TBC project or not cannot be ascertained as date of diagnosis was missing. cfollow-up period from diagnosis ranged from 6 months to 2 years.

**Fig 3:**
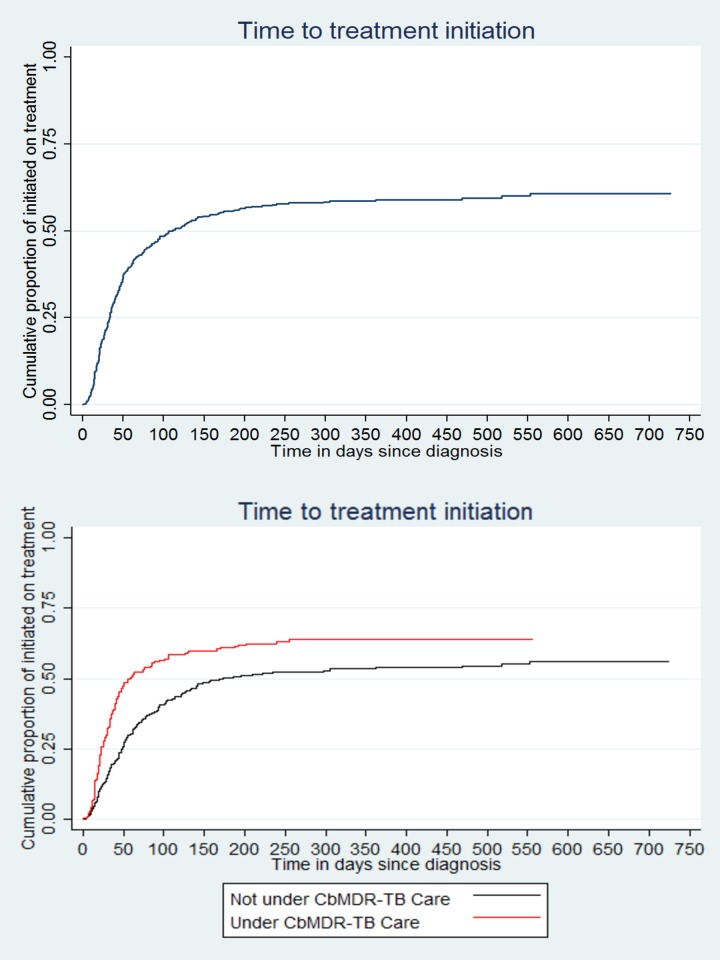
Time to treatment initiation among patients diagnosed with MDR-TB (overall and stratified by CBMDR-TBC status) between January 2015 and June 2016 in 33 CBMDR-TBC project supported townships in upper Myanmar. *MDR-TB-Multidrug resistance tuberculosis, CBMDR-TBC- Community based multidrug resistance tuberculosis care project.

The number (% (0.95 CI)) of patients initiated on treatment among those “not receiving support (n = 228)” and “receiving support (n = 208)” was 127 [56(49, 62)] and 132 [64 (57, 70)] respectively **(Fig 2)**. The cumulative proportion initiated on treatment with time is summarized in **Fig 3**. Median (IQR) time to initiate treatment was 38 (20, 76) days: 31 (18, 50) among patients “receiving support” and 50 (26, 101) among patients “not receiving support”. Overall, incidence rate (0.95 CI) of treatment initiation was 2.6 (2.3, 3.0) per 1000 person-days of follow-up: 4.2 (3.5, 5.0) among patients “receiving support” and 1.9 (1.6, 2.2) among patients “not receiving support”.

### Effect of CBMDR-TBC project on treatment initiation

Age, sex, region, previous history of TB, HIV status and CBMDR-TBC status were included in the cox regression model. Distance was not included as its unadjusted p value was >0.2. As CBMDR-TBC status was not fulfilling the proportional hazards assumption, we modelled for time varying association before and after 120 days of follow-up because the observed and predicted survival probabilities were crossing over at 120 days **(S1 Fig)** [14]. For CBMDR-TBC, adjusted HR at follow-up between 0 and 120 days and adjusted HR at follow-up after 120 days (interaction between CBMDR-TBC and time>120) is also presented **(Table 4).**

**Table 4:** Factors associated with treatment initiation among patients diagnosed with MDR-TB between January 2015 and June 2016 in thirty three CB MDR-TBC project townships in Myanmar.

			Treatment		HR	*aHR
Characteristics		Total	initiation		(0.95 CI)	(0.95 CI)
		N	n	(%)		
**Total**		456	261	(57)	-	-
Age (year)	< 15	4	1	(25)	0.5 (0.1,3.6)	0.5 (0.1,4.0)
	15–34	187	118	(63)	1.6 (1.1,2.3)	**1.7 (1.2,2.5)^**
	35–54	178	103	(58)	1.4 (1.0, 2.0)	**1.5 (1.03,2.2)^**
	≥55	87	39	(45)	**Ref**	**Ref**
Sex	Male	305	129	(42)	0.99 (0.8, 1.3)	1.0 (0.8,1.3)
	Female	151	66	(44)	**Ref**	**Ref**
Patient residence	Mandalay	277	159	(57)	1.2 (0.8,1.7)	1.2 (0.8, 1.8)
state/region	Magway	41	26	(63)	1.4 (0.9,2.4)	1.3 (0.8, 2.2)
	Sagaing	62	33	(53)	**Ref**	**Ref**
	Northern Shan	49	26	(53)	1.1 (0.7,1.9)	1.1 (0.6, 1.8)
	Southern Shan	27	17	(63)	1.3(0.7,2.4)	1.2 (0.6,2.1)
Previously treated	Yes	359	232	(65)	2.2 (1.5,3.3)	**2.2 (1.5, 3.4)^**
TB	No	86	29	(34)	**Ref**	**Ref**
	Unknown	11	0	(0)	-	-
HIV status	Reactive	74	30	(41)	**Ref**	Ref
	Unknown	127	75	(59)	1.7 (1.1,2.6)	**1.6 (1.0,2.6)^**
	Non-reactive	255	156	(61)	1.8 (1.2,2.7)	**1.6 (1.1,2.4)^**
Distance of patient	0 km	114	64	(56)	1.0 (0.7,1.5)	-
township from	<100 km	212	125	(59)	1.1 (0.8, 1.5)	-
treatment facility	≥100 km	130	72	(55)	**Ref**	-
Receiving support	**At time 0–120**					
project (exp1- main)	Yes	208	132	(64)	1.5 (1.1,1.9)	**1.8 (1.3,2.3)^**
	No	228	127	(56)	**Ref**	**Ref**
tvc (interaction with	**At time >120**					
_t>120)**	Yes	85	10	(12)	**-**	**0.4 (0.2–0.9)^**
	No	127	26	(21)	**-**	**Ref**

HR- Hazard Ratio; aHR- Adjusted HR; CI–confidence interval; ref- reference; CBMDR-TBC—community-based multi-drug resistant tuberculosis care project

*aHR calculated using Cox regression (enter method—complete case analysis): age, sex, CBMDR-TBC status and variables with unadjusted p<0.2 were included in the regression model

**Interaction term between time>120 days and CBMDR-TB status, proportional hazards assumption not met, the stcoxph curve showed that around 120 days there was a cut off for time varying association. We used tvc function in STATA to model time varying associations.

^p<0.05

After adjusting potential confounders, during follow-up between 0 and 120 days, patients “receiving support” had 80% higher chance of initiating treatment [aHR (0.95 CI): 1.8 (1.3, 2.3)] when compared to patients “not receiving support”. This effect seemed to reverse (60% lower chance) after 120 days [aHR (0.95 CI): 0.4 (0.2, 0.9)]. In addition, patients aged between 15 and 54 years (compared to age group >55 years), patients who had previous history of TB (when compared to no history) and patients with HIV negative or unknown (when compared to HIV positive) were independent predictors of treatment initiation throughout the follow-up period **(Table 4).**

## Discussion

### Summary of key findings

This is the first study done worldwide to study the effect of a support package at the community level (CBMDR-TBC in our case) on time to treatment initiation among patients diagnosed with MDR-TB in the context of domiciliary care through PMDT. Three out of five patients diagnosed with MDR-TB got initiated on treatment while other underwent pre-treatment loss to follow-up. The time to initiate treatment was long. Within four months of diagnosing MDR-TB, the CBMDR-TBC project’s support increased treatment initiation and shortened the time to treatment initiation, hence, reducing disease transmission.

### Strengths and limitations

There are several strengths of this study. We covered 10% (33 townships out of 330 townships) of total townships in Myanmar. Hence, we included sufficiently large number of patients with MDR-TB in the study. Second, reversal of hazards (after 120 days in our case) is a known epidemiological phenomenon where cohorts are followed up for a long periods of time [15]. We therefore modelled for time varying effect of CBMDR-TBC status in our time to event analysis. In the absence of this modeling, we ran the risk of getting a null effect estimate. Third, the study involved use of routine programmatic data; therefore, our findings reflect the ground reality.

However, there are some limitations in this study. First, diagnosis dates were missing for some of the patients and exposure ascertainment could not be done in 4% of the patients. Second, we do not know what happened to the patients who did not start on treatment. Studies have shown that about 40% of patient who recorded as not initiated treatment at the treatment centers were identified as out-migration to other area or cannot be traced due to wrong address and about 10% as death [16,17]. Therefore, the diagnosed patients who did not start treatment from our study could also have either died or taken treatment in DR-TB centers outside the 33 project townships. Third, information on other patient level data (extent of exposure to CBMDR-TBC in the form of number of nurse /volunteer visits) and programmatic / health system level factors was not available as this was not collected routinely within the programme. There might be some measurement errors that are inherent to operational research. Fourth, the date of project initiation was at the township level. However, we do not think that this would induce any clustering as all patients (previously diagnosed and newly diagnosed) were provided services once the project was implemented in a township. Finally, we did not consider the distance from actual patient residence. Hence, if the township was large, there might be large margin of error depending where someone lived in that township. However, this error was not expected to vary differentially among the CBMDR-TBC groups (‘receiving support’ and ‘not receiving support’).

### Interpretation of findings

Overall, the treatment initiation proportion was low: two-fifths were not initiated on treatment. Considering the analysis was time to event, the follow-up time in our cohort was between six months and two years. The treatment initiation may be even lower if we applied constant follow-up time for each patient (say 6 months). The proportion of patients not initiated on treatment was lower than the study done in South Africa. However, it was higher than the global estimates (5%) and findings in Bangladesh and India [1–4]. One of the reasons for this could be that decentralization of treatment form two main DR-TB centers of Myanmar (Yangon and Mandalay) to district-level DR-TB centers began in 2013 and was completed in March 2016. Therefore, MDR-TB patients diagnosed in 2015 and 2016 included in our study may have experienced difficulties to start treatment especially for patients from townships outside Mandalay.

According to NTP MDR-TB management guidelines (2016), all diagnosed MDR- TB patients should be put on treatment within 14 days after diagnosis. However, this study revealed that only a small percentage (14%) of diagnosed patients could start treatment within 14 days. This delay in the presence of Xpert MTB/Rif which takes only two hours to diagnose RR-TB is unacceptable as introduction of rapid molecular diagnostic tests has worldwide shown a reduction in time to treatment initiation. [3,6]

CBMDR-TBC project had a positive effect on improving patients’ treatment initiation in the first four months after diagnosis: it not only improved the proportion initiated on treatment but also reduced the time to treatment initiation. We did not find any other study worldwide, which studied the effect of a support package to improve treatment initiation among patients receiving domiciliary PMDT services. There are studies that have compared community-based care with facility-based care and documented earlier treatment initiation [18]. However, this cannot be compared with our study as the comparison in our study was between domiciliary care under PMDT with and without a support package from CBMDR-TBC project. A study from India looked at the effect of implementation of recommendations from an operational research on pre-treatment loss to follow-up and time to initiate treatment. However, this was a before and after design with no control arm and the number of diagnosed patients were less than at each given study period [19].

There are possible reasons within CBMDR-TBC project which could be responsible for the improvement in treatment initiation. First, we have reasons to believe that the PMDT guidelines to reduce pre-treatment loss to follow-up would have been more effectively implemented among patients “receiving support”. The project focal nurse was exclusively assigned to implement PMDT guidelines and specific components of the CBMDR-TBC project. The project focal nurse also coordinated with the MDR-TB treatment center for pre-treatment baseline investigations of the patient **(Tables 1 and 2).** Patients “not receiving support” from the CBMDR-TBC project received services only from BHS who delivers not only TB activities but also other health activities.

Second, patients “receiving support” received 30 USD per month (max 4 months) in the pre-treatment period which is not provided under PMDT. This may reduce the financial burden on the patient for their visit to DR-TB center for pre-treatment evaluation.

Despite this, there was scope for improvement among those receiving CBMDR-TBC support as well. Low treatment initiation (64%) in patients “receiving support” from the CBMDR-TBC project may be due to existing systemic issues which require improvement and were beyond the scope of CBMDR-TBC project. Other factors affecting treatment initiation are timely result feedback to patients, negative perceptions of the adverse effects of MDRTB treatment by the patients, lack of human resources who can provide timely referral and manage those adverse effects properly, funding limitations and limited infrastructure for MDRTB care and service [20]. PMDT along with CBMDR-TBC project needs to focus the high risk groups for pre-treatment loss to follow-up identified in our study (HIV positive, new patient, old people) [4].

### Policy implications

There are many policy implications of this study. First, the PMDT in Myanmar needs to urgently take steps to reduce pre-treatment loss to follow-up and time to treatment initiation. Both PMDT and CBMDR-TBC project may consider updating the township-level presumptive MDR-TB register from time to time. This will enable tracking each patient once diagnosed. An indicator of pre-treatment loss to follow-up should be made in the quarterly reports of PMDT and monthly reports of CBMDR-TBC.

Second, the PMDT and CBMDR-TBC may consider the use of innovative ways to communicate Xpert MTB/Rif results to the township TB center. This may include short messaging services, emails or using internet-based mobile applications. Third, the existing pre-treatment evaluation process needs to be simplified and streamlined.

Fourth, we recommend expansion of the support from CBMDR-TBC to all townships and it needs to systematically implemented and monitored. Currently, The Union is one of the non-Government organizations supporting the PMDT in providing this care.

Fifth, there is a need for further research into what happens to patients who do not initiate treatment. We also recommend qualitative systematic enquiry to study patient and health-system related risk factors for pre-treatment loss to follow-up and enablers for treatment initiation among patient receiving support from CBMDR-TBC project.

## Conclusion

Receiving support from CBMDR-TB care project improved treatment initiation among patients diagnosed with MDR-TB within four months of diagnosis in Myanmar. However, the treatment initiation among patients “receiving support” was still far behind WHO End TB targets [21]. Improved tracking of patients diagnosed with MDR-TB with special focus on HIV positive, new TB patient and old people are urgently required.

## Supporting information

S1 Fig Assessment of proportional hazards assumption for treatment initiation by plotting the estimated survival curves obtained using Cox model and Kaplan-Meier estimates, stratified by CBMDR-TBC status. *Exp1 variables categorized as (Yes) “receiving CBMDR-TBC support”; (No) “not receiving CBMDR-TBC support”.
CBMDR-TBC—community-based multi-drug resistant tuberculosis care project.
In “not receiving CBMDR-TBC support” group, before time = 120 days (approx.), predicted values are an underestimate of the observed values, while after time = 120 days (approx.), predicted values are an overestimate of the observed values.
In “receiving CBMDR-TBC support” group, before time = 120 days (approx.), predicted values are an overestimate of the observed values, while after time = 120 days (approx.), predicted values are an underestimate of observed values.
(TIF)

S1 Annex Dataset and programme file used for analysis. (ZIP)
