# Supplementary figures and images for: Community-based MDR-TB care project improves treatment initiation in patients diagnosed with MDR-TB in Myanmar
Source: PLoS One. 2018 Mar 29;13(3):e0194087. doi: 10.1371/journal.pone.0194087 (PMC5875775; doi:10.1371/journal.pone.0194087)

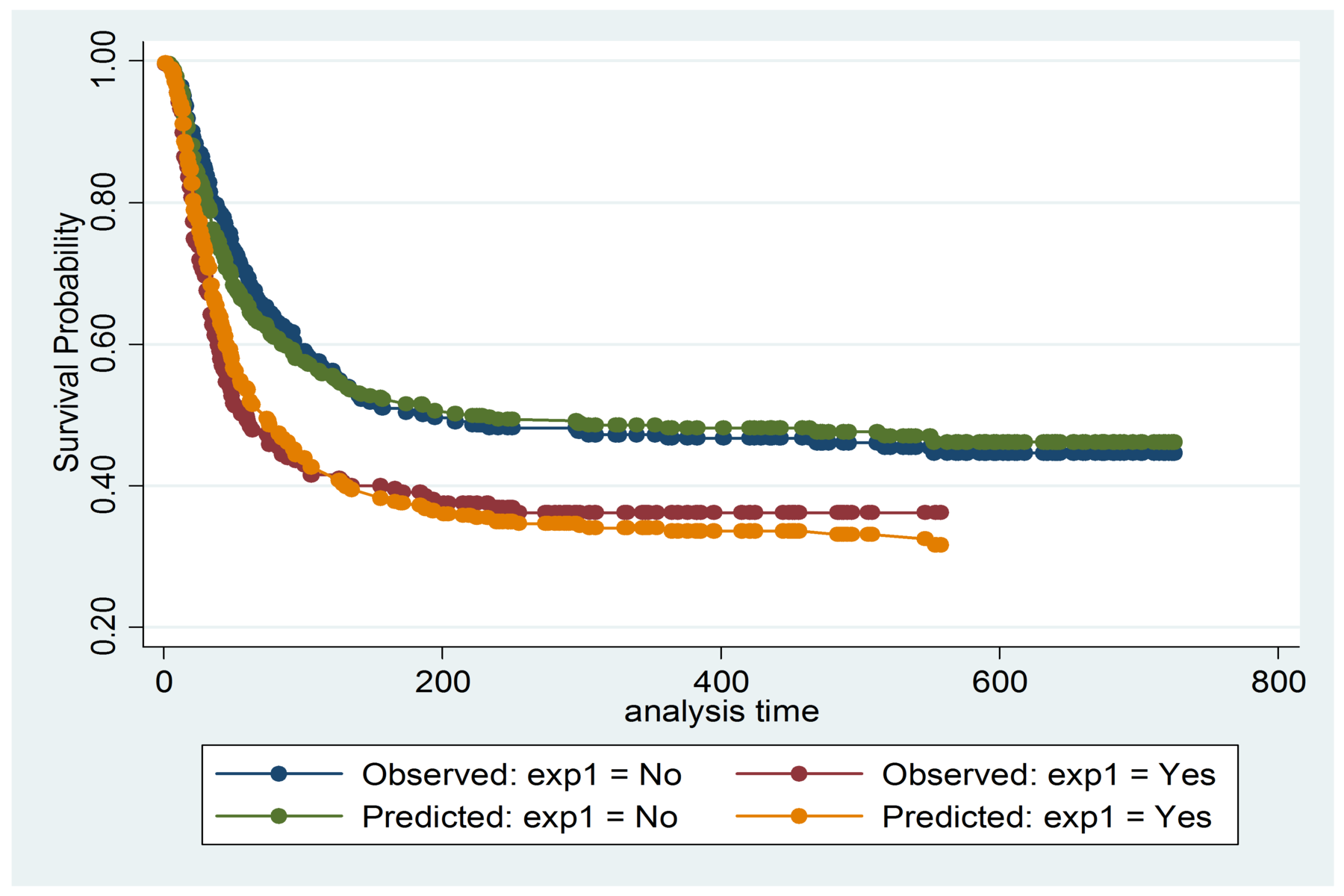

Supplement: S1 Fig — *Exp1 variables categorized as (Yes) “receiving CBMDR-TBC support”; (No) “not receiving CBMDR-TBC support”. CBMDR-TBC—community-based multi-drug resistant tuberculosis care project. In “not receiving CBMDR-TBC support” group, before time = 120 days (approx.), predicted values are an underestimate of the observed values, while after time = 120 days (approx.), predicted values are an overestimate of the observed values. In “receiving CBMDR-TBC support” group, before time = 120 days (approx.), predicted values are an overestimate of the observed values, while after time = 120 days (approx.), predicted values are an underestimate of observed values. (TIF) [file pone.0194087.s001.tif]
